# Vaping in Pregnancy: Unraveling Molecular Drivers of Preeclampsia and Fetal Growth Restriction

**DOI:** 10.3390/ijms262010009

**Published:** 2025-10-15

**Authors:** Archarlie Chou, Olivia Hiatt, Benjamin Davidson, Paul R. Reynolds, Brett E. Pickett, Juan A. Arroyo

**Affiliations:** 1 Department of Microbiology and Molecular Biology, Brigham Young University, Provo, UT 84602, USA; 2 Department of Cell Biology and Physiology, Brigham Young University, 3052 LSB, Provo, UT 84602, USA

**Keywords:** preeclampsia, intrauterine growth restriction, electronic cigarette, RNA-sequencing, placental dysfunction

## Abstract

Preeclampsia (PE) and intrauterine growth restriction (IUGR) are major pregnancy complications that are linked to placental dysfunction and environmental stimulation such as the use of electronic cigarettes (eCig). This study investigates the molecular impacts of timed eCig exposure in a C57BL/6 mouse model of PE and IUGR using bulk RNA-sequencing of placental tissues. Pregnant mice were exposed to eCig vapor via nose-only system starting at embryonic day 12.5 (eCig-6d, before spiral artery (SA) invasion) or 14.5 (eCig-4d, after SA invasion) until E18.5 (necropsy), with healthy controls exposed to room air (*n* = 6/group). The eCig-4d group developed PE, whereas the eCig-6d group developed both PE and IUGR. RNA-seq analysis revealed 429 differentially expressed genes (DEGs) in eCig-4d (IUGR-like) group and 64 DEGs in eCig-6d (PE + IUGR-like) group compared to controls. Pathway and gene network analyses indicated that eCig-4d exposure activated NF-κB–driven inflammation, suppressed ECM organization and collagen biosynthesis, and downregulated vasoactive genes/mitochondrial-associated genes (*NOS1/2*), accompanied by impaired complement initiation and reduced both macrophage and monocyte signals. Similarly, eCig-6d exposure led to downregulation of complement-associated genes and granule-related components, possibly implicating weakened neutrophil responsiveness and compromised inflammatory resolution at the maternal–fetal interface. Our findings align with prior studies on physiological dysfunctions in PE and IUGR, while also providing novel insights into the temporally specific cellular responses induced by eCig exposure.

## 1. Introduction

Preeclampsia (PE) is a severe hypertensive disorder characterized by new-onset hypertension after 20 weeks of gestation, accompanied by proteinuria and organ dysfunction, posing great risks to both maternal and fetal health [1]. Affecting 2–8% of pregnancies worldwide, PE contributes to over 45,000 maternal deaths annually [2,3]. The pathophysiology of PE is multifaceted, involving defective spiral artery (SA) invasion, where endothelial trophoblast invasion fails to adequately transform uterine spiral arteries into maternal spiral arteries, resulting in placental hypoperfusion and hyper-oxidative stress [4,5]. Frequently co-occurring with PE, intrauterine growth restriction (IUGR) is also a prevalent pregnancy complication. Typical symptoms of IUGR involve impaired fetal growth due to inadequate nutrient and oxygen supply, primarily from placental insufficiency [6]. IUGR affects approximately 17% of liveborn pregnancies, heightening the risk of long-term cardiovascular and metabolic complications in offspring [4]. Given the vulnerability of placental development in PE and IUGR, emerging maternal lifestyle factors such as electronic cigarette (eCig) usage warrant attention.

eCig, perceived as “safer” alternatives to traditional cigarettes, have quickly gained popularity among young adults [7]. For pregnant mothers, eCig usage presents a substantial threat to both placental and fetal health [8]. Most eCigs contain nicotine, which passes through the placenta to the fetus, subjecting the fetus to higher nicotine levels than the mother [9,10]. Furthermore, inhaling eCigs without nicotine additives still leads to reduced prolactin levels and impaired vasodilation in the middle cerebral artery, showing the independence of toxicity in eCig smoke contents [11,12]. Meta-analyses also link eCig use in pregnancy to adverse outcomes, including preterm birth, low birth weight, and stillbirth [13,14]. These outcomes are mechanistically linked to PE and IUGR through eCig-induced placental dysfunction, including impaired second wave of trophoblast invasion, increased oxidative stress, and vascular remodeling defects, which mirror the hypoperfusion seen in these disorders [12,15,16,17]. eCig aerosols can also disrupt endothelial function and promote inflammation in placental tissues, thereby exacerbating shallow SA invasion and restricting fetal nutrient supply [18,19]. Animal models also demonstrated that exposure to eCig aerosols in mice induced PE-like symptoms, with their outcomes varying by the timing of exposure relative to SA invasion [18].

Common physiological observations in PE or IUGR include elevated collagen deposition. Collagen type I and III are the building blocks for extracellular matrices (ECM) that organize histological architecture [20]. However, elevated collagen I could induce fibrosis in the uterine decidua, creating barriers for SA invasions. Collagen I-induced PE is well recognized in human and rat models [21]. However, decreased collagen expression could also be associated with PE as a compensatory response to such physiological assaults [22]. We focused on collagen pathways because of their central role in ECM remodeling during placentation. We also examined complement pathways, which are essential for immune tolerance at the maternal–fetal interface. Dysregulation of either pathway has been implicated in defective SA invasion and placental insufficiency in PE/IUGR models [23,24], often compounded by mitochondrial dysfunction from oxidative stress, as seen in both smoking and vaping exposures [25]. 

To expand on this, the complement system represents a cascade of proteins that enables the innate immune system to respond to pathogens. Within the classical pathway, the first necessary protein is C1, which initiates the inflammatory response [26]. The main functional component of C1 (C1q) comprises six chains: two sets of A and B chains and two C-chains. The main components of these sub-chains are also collagen proteins [27,28]. In one study, C57BL/6 C1q-deficient mice were found to be at high risk of fetal resorptions and reduced fetal weight [29]. However, in another study, inhibition of complement activation in C3, another downstream protein in the complement cascade, was shown to have protective effects against PE in mice [30].

Beyond complement, other immune cell populations also contribute to the inflammatory conditions in PE and IUGR. Macrophages and monocytes promote pro-inflammatory environments, while dendritic cells modulate T-cell responses [31,32]. In IUGR pregnancies, intermediate monocyte counts are increased [33,34]. To uncover the genetic basis of these observations, we employed RNA-seq.

RNA-seq is a common technique for gaining molecular insights by characterizing transcriptomic profiles. Researchers often construct a gene regulatory network (GRN) to investigate how genes regulate each other, thereby representing the relationships between transcription factors and their targeted genes [35]. By analyzing this network, we can begin to deconvolute underlying regulatory sub-networks. Sub-networks can be analyzed with enrichment techniques such as Gene Ontology (GO) analysis that defines biological processes, molecular functions, and cellular components; as well as pathway enrichment analysis using databases like KEGG and Reactome [36,37,38]. Finally, besides GRN analysis, immune cell deconvolution is a widely used approach to measure the immune cell signals based on gene expression signatures [39]. Immune cell deconvolution via CIBERSORTx was selected for its robustness in estimating cell-type proportions from bulk RNA-seq data using validated signature matrices, particularly suited for heterogeneous tissues like the placenta, where direct cell isolation is challenging [40].

**Figure 1:**
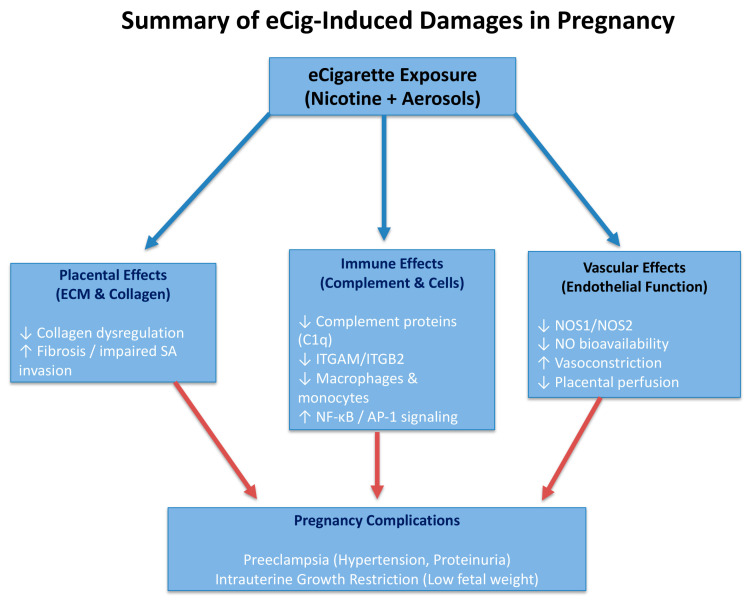
Schematic summary of eCig exposure-induced placental and fetal damage. eCig aerosols, including both nicotine and non-nicotine components, disrupt multiple biological systems involved in placentation. These include impaired SA invasion and oxidative stress in the vascular system; immune dysregulation via altered complement signaling, dendritic cells, and pro-inflammatory macrophage/monocyte activity; and ECM remodeling defects marked by dysregulated collagen expression and fibrosis.

In this study, we employed a well-characterized C57BL/6 mouse model of preeclampsia and intrauterine growth restriction, which has been validated in our recent publications (Figure 1) [18,41]. This model allows for controlled, time-dependent exposure to eCig vapor relative to SA invasion, a critical developmental milestone in placentation. Specifically, initiating exposure at embryonic day 12.5 (prior to SA invasion) results in combined PE and IUGR disease phenotypes, whereas exposure at embryonic day 14.5 (after SA has begun) predominantly induces IUGR features. Importantly, the vascular, immune, and ECM alterations observed in this model parallel pathological processes described in human pregnancy, showing its predictive value and translational relevance for understanding vaping-associated risks in maternal–fetal health. To avoid confusion, we clarify the terminology used throughout this work. The condition referred to as eCig4-induced IUGR model is used interchangeably with eCig4 days exposure, eCig4 days, eCig4 vs. Control, and eCig4 vs. Healthy. Similarly, eCig6-induced IUGR + PE model may also be described as eCig6 days exposure, eCig6 days, eCig6 vs. Control, and eCig6 vs. Healthy. While these labels differ, they all describe the same comparison, and the choice of term reflects the context of each analysis.

This study aims to better characterize the underlying intracellular transcriptional response to eCig exposure at two time points, associating these in vivo observations with their biological processes and pathways. Comparing these time points is significant as it reflects potential human exposure windows during placentation, allowing identification of time-dependent molecular drivers and potential therapeutic targets for repurposing in PE/IUGR [42]. Furthermore, this work underscores the harmful effects of vaping in pregnancy, despite its perceived safety.

## 2. Results

### 2.1. Blood Pressure, Proteinuria, and Weight Assessments

**Figure 2:**
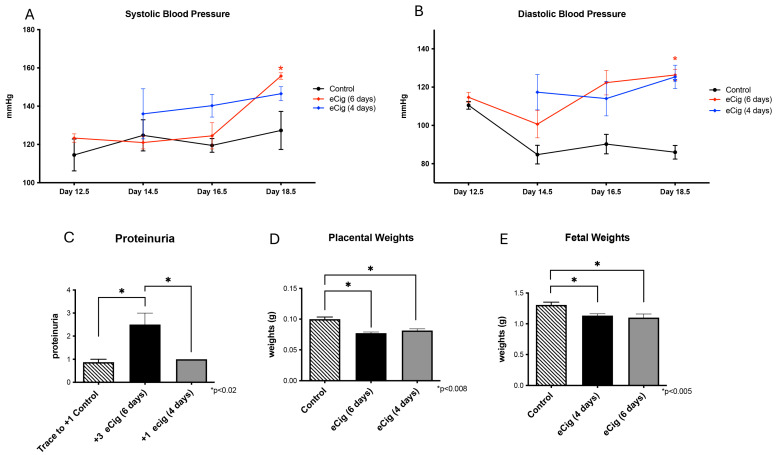
Blood pressure, proteinuria, and weight after eCig exposure. (**A**) The effects of four- and six-day eCig exposure on systolic blood pressure. (**B**) The effects of four- and six-day eCig exposure on diastolic blood pressure * *p* < 0.05. (**C**) The effects of four- and six-day eCig exposure on proteinuria. (**D**) The effects of four- and six-day eCig exposure on the placenta. (**E**) The effects of four- and six-day eCig exposure on fetal weights.

Elevated blood pressure is a key indicator of PE. Blood pressure measurements were collected to evaluate the impact of eCig exposure. At necropsy, both systolic and diastolic blood pressures were significantly elevated (*p* < 0.04) in mice exposed to eCig for six days (Figure 2A,B). In contrast, four days of eCig exposure significantly increased diastolic blood pressure (Figure 2A,B). Proteinuria, which is another critical marker of PE, showed a significant 2.5-fold increase (*p* < 0.004) after six days of eCig exposure (Figure 2C). Reduced placental and fetal weights are characteristic of IUGR. To assess this, we measured changes in placental and fetal weights following eCig exposure. Both six-day (1.3-fold, *p* < 0.0001; 1.2-fold, *p* < 0.005) and four-day (1.2-fold, *p* < 0.0007; 1.2-fold, *p* < 0.005) eCig treatments led to significant reductions in placental and fetal weights, respectively (Figure 2D,E).

**Figure 3:**
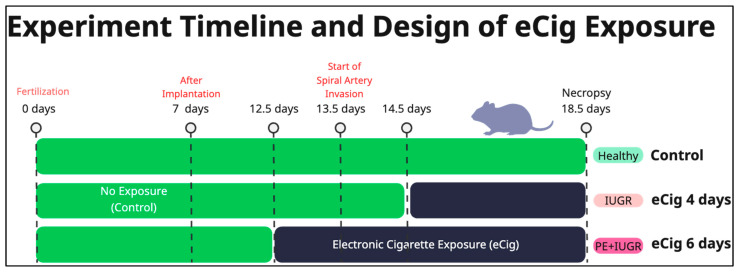
Overview of Experiment Design. Critical time points are labeled—days 0: fertilization, days 7: implantation, day 12.5: eCig6d inhalation begins, 13.5 spiral artery remolding starts, 14.5: eCig4d inhalation begins, day 18.5: Necropsy. The resulting symptoms are shown along with the experimental conditions compared to the control.

From the elevated systolic and diastolic blood pressure, we concluded that 6 days of eCig inhalation during gestation in mice results in both PE and IUGR-like symptoms, whereas 4 days of eCig inhalation results in only IUGR-like symptoms (Figure 3).

### 2.2. Differential Gene Expression Analysis

**Figure 4:**
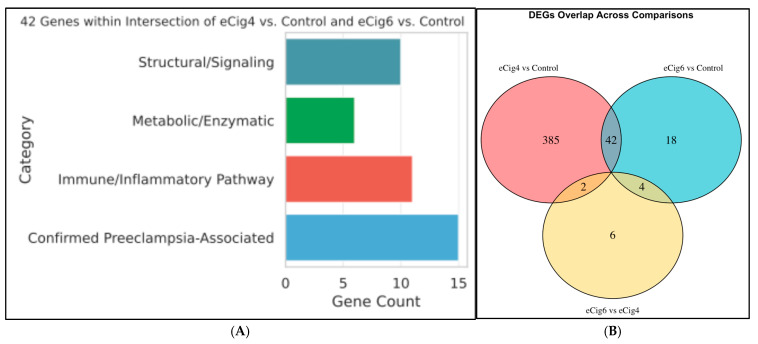

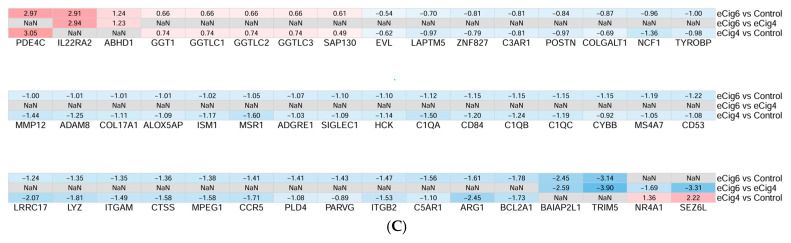
Shared Gene Expression Patterns and Functional Categories in eCig vs. Control Comparisons (**A**) Venn diagram of DEGs identified from the three different comparisons. (**B**) 42 Genes within Intersection of eCig4 vs. Control and eCig6 vs. Control: Distribution of genes by functional categories from literature search in relation to PE. (**C**) Direct comparison of DEG expression (salmon/pink: up-regulation; blue/light-blue: down-regulation) across all three groups of experiment design. Genes (columns) and design (rows, identical in every strip) show the direction of regulation in the overlapped genes.

To understand the genes contributing to this phenomenon, we identified differentially expressed genes (DEGs) across three comparisons (Figure 4A). These groups are (1) eCig4-induced PE model (eCig4 vs. Healthy), (2) eCig6-induced PE + IUGR model (eCig6 vs. Healthy), and (3) eCig6 vs. eCig4.

Interestingly, mice exposed to eCig smoke for six days (eCig6 vs. Control) had far fewer significant DEGs (64) compared to mice exposed for four days (eCig4 vs. Control) (429 DEGs). (Figure 4B) While both exposure durations resulted in IUGR, our analysis suggests that a different set of gene products contributes to the development of IUGR. Most significant DEGs identified in comparison eCig6 vs. Control did not show up in that of eCig4 vs. Control comparison, and vice versa, suggesting that the longer exposure time does not simply amplify the eCig exposure effects, but triggers a distinct transcriptomic response. All overlapping genes between the eCig4-induced IUGR model and the eCig6-induced IUGR + PE model, compared to healthy controls, were up- or downregulated in the same direction (Figure 4C). These shared DEGs, including complement-related genes (*C1QA-C*) and immune signaling markers (*ITGAM*, *ITGB2*), indicate immune suppression akin to that observed in traditional smoking, with additional mitochondrial dysfunction implied by NOS downregulation [24,43,44].

Of the 42 genes (Figure 4B) shared between the two models (eCig4-induced IUGR and eCig6-induced IUGR + PE), several known PE diagnostic markers (COL17A1, TYROBP, HCK, MMP12, SIGLEC1) were consistently downregulated. Complement system-related genes were also suppressed, including *C1QA-C* (components of the C1q complex) and *ITGAM*, *ITGB2*, *C3AR1*, and *C5AR1* (complement receptors). Markers associated with inflammatory responses and M2 polarization (ARG1, MSR1, MS4A7, ADGRE1, MPEG1), along with membrane proteins involved in immune cell signaling (CD84, CD53), were all downregulated. Additionally, 12 DEGs were identified when comparing the 4- to 6-day eCig exposures. (Figure 4A) *IL22RA2* was upregulated, while *TRIM5*, *TRIM34*, and *ENOS* were downregulated. *IL22RA2* (log_2_FC = 2.94, FDR = 0.011) is an inflammatory marker, *TRIM5* (log_2_FC = −3.90, FDR = 2.91 × 10^−9^) is an antiviral restriction factor, and TRIM34 (log_2_FC = −1.45, FDR = 1 × 10^−5^) is involved in interferon signaling. *ENOS* (log_2_FC = −1.90, FDR = 0.016) is a well-characterized PE biomarker responsive to hypoxia.

### 2.3. eCig4-Induced IUGR Model DEG Analysis

**Figure 5:**
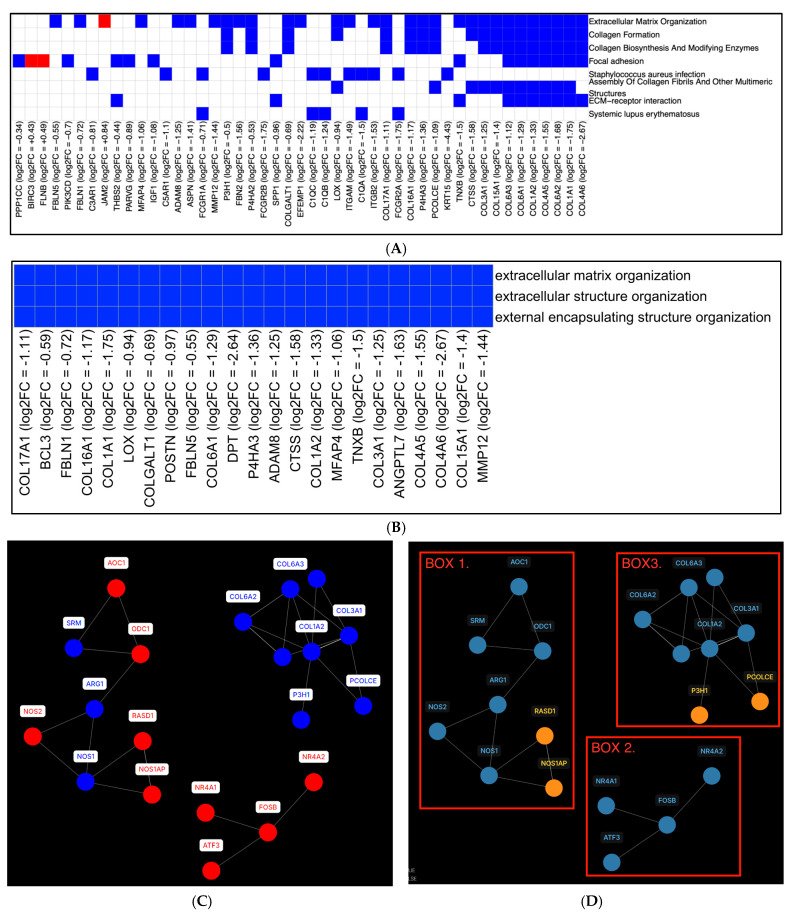
Differential Gene Expression and Regulatory Network Analysis in the eCig4-Induced PE Model. (**A**) SPIA pathway enrichment heatmap, showing gene expression changes (red: upregulated; blue: downregulated) across pathways across genes. The log₂ fold changes are concatenated with the gene names. (**B**) GO term enrichment heatmap, annotated with expression levels (blue: downregulated) for each gene. The log₂ fold changes are concatenated with the gene names. (**C**) GRN for the eCig4-induced PE model: edges (bi−directional influences) represent protein interactions; nodes (gene products) are colored by direction of differential expression (red: upregulated; blue: downregulated). (**D**) GRN annotated with disease-associated gene sets (blue: literature-recorded associations with PE; orange: unrecorded). Cluster with red boxes for identification.

Exposure to eCig4, starting after SA invasion, led to inhibition of multiple collagen formation and ECM organization pathways (Figure 5A,B) (Table 1). Reduced expression was also seen in collagen genes *COL1A1*, *COL1A2*, *COL3A1*, *COL4A5*, *COL4A6*, *COL6A1*, *COL6A3*, and *COL15A1*, along with complement system genes *C1QA-C*.

To characterize the cellular signals after four days of eCig exposure compared to healthy controls, we analyzed the GRN in the eCig4-induced PE model (Figure 5C). Gene to gene interactions were first inferred and assigned weighted scores via the KBoost algorithm, which we then used to identify the most influential pairs of interactions. These high-confidence interactions were subsequently mapped onto STRING protein interaction data to construct the final GRN. The GRN was organized into 15 distinct subnetworks. One subnetwork contained transcription factors that strongly influenced each other and eventually mutually upregulated each other. (Figure 5D, Box 2). Among them, NR4A1 and NR4A2 are nuclear receptor transcription factors, with NR4A members being particularly involved in inflammatory regulation. *FOSB*, which encodes a leucine-zipper transcription factor, contributes to AP-1 complex formation that plays a critical role in inflammation. The above genes are well recognized in PE pathogenesis. Furthermore, in another subnetwork (Figure 5D, Box 1), we observed vasoactive regulation genes, including *NOS1*, *NOS2*, *NOS1AP*, and *ARG1*, which strongly regulate nitric oxide production. *ARG* and *NOS2* were downregulated, while *NOS1* and *NOS1AP* were upregulated. The downregulation of collagen genes was again evident in this subnetwork. (Figure 5D, Box 3). This network also suggests mitochondrial impairment via NOS1/2 dysregulation, leading to oxidative stress - effects shared with cigarette smoking but potentially less severe in vaping [25,45]. Additionally, vaping with nicotine may enhance thrombus formation, potentially exacerbating vascular dysfunction [45].

In this network, we also identified markers of immune cells. The *ITGB2*, *ITGAM*, *HCK*, and *TYROBP* genes (all downregulated) are expressed in both monocytes and macrophages and are associated with M2 polarization. Within the complement pathway, *C5AR1* (downregulated) is highly expressed in dendritic cells, where its suppression may impair T-cell activation.

### 2.4. eCig6-Induced IUGR + PE Model

**Figure 6:**
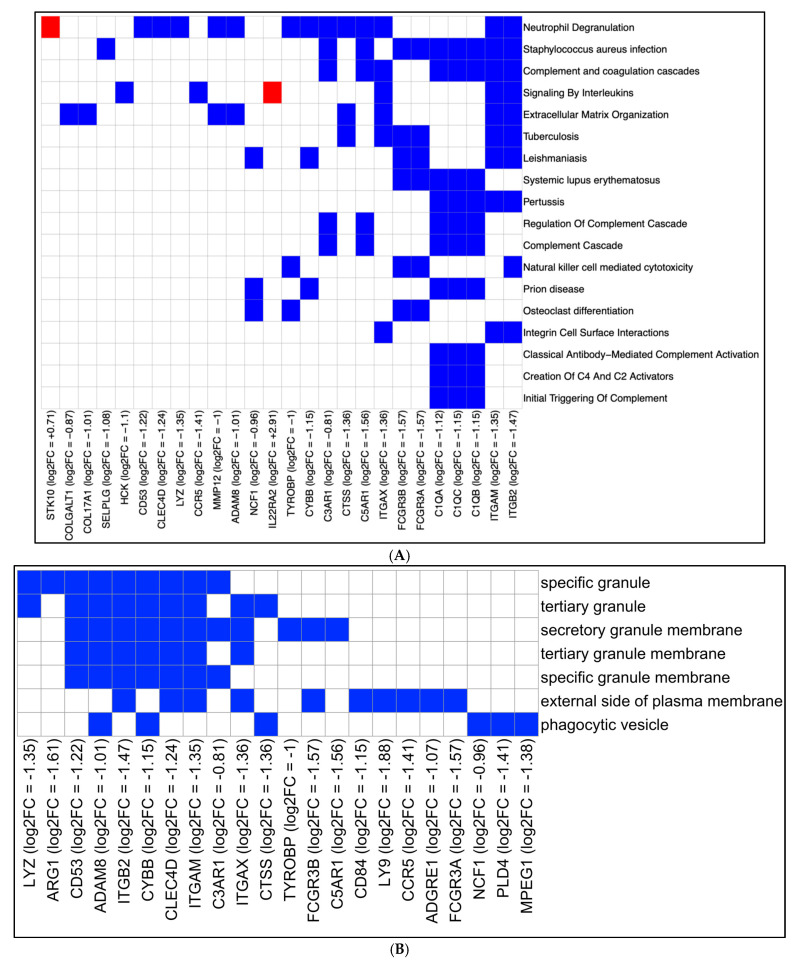

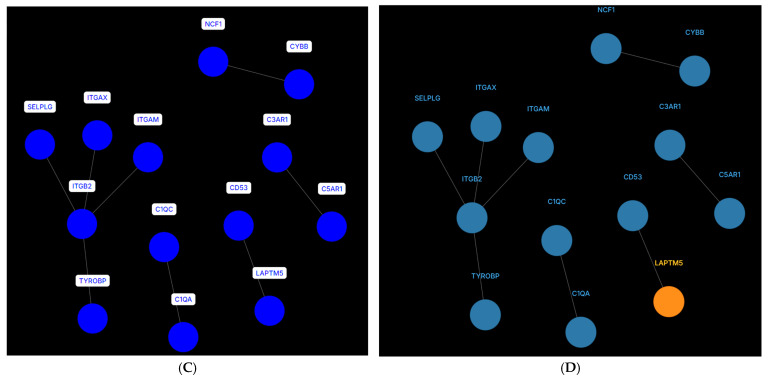
Differential Gene Expression and Regulatory Network Analysis in the eCig6−induced IUGR + PE Model (**A**) SPIA pathway enrichment heatmap, showing gene expression changes (red: upregulated; blue: downregulated) across pathways across genes. The log₂ fold changes are concatenated with the gene names (**B**) GO term enrichment heatmap, annotated with expression levels (red: upregulated; blue: downregulated) for each gene. The log₂ fold changes are concatenated with the gene names. (**C**) GRN for the eCig6−induced IUGR + PE model: edges (bi−directional influences) represent protein–protein interaction; nodes (genes) are colored by direction of differential expression (red: upregulated (none here); blue: downregulated). (**D**) GRN annotated with disease-associated gene sets (blue: literature-recorded associations with PE; orange: unrecorded).

In the eCig6-induced IUGR + PE model (Figure 6A; Table 2), pathways related to complement activation and infection were significantly enriched and predicted to be inhibited. Consistent with earlier findings, the core complement genes *C1QA-C* were downregulated. This suppression, along with reduced expression of integrins such as *ITGAM* and *ITGB2* (integrin), likely contributed to the enrichment of infection-related pathways, including those associated with prion disease and Staphylococcus aureus infection. Interestingly, neutrophil degranulation was also predicted to be deactivated, suggesting potential impairment in innate immune responses. Supporting this, GO enrichment analysis (Figure 6B) revealed widespread downregulation of genes involved in granule formation and transportation.

In the eCig6-induced IUGR + PE GRN, all genes were downregulated, and notably, none were transcription factors. Within the integrin subunit cluster, we observed potentially strong interactions among ITGB2, ITGAM, and ITGAX. ITGB2 and ITGAM are critical for immune cell adhesion, while ITGAX functions as a subunit of complement receptor 4 (CR4). Similarly, both C5AR1 and C3AR1 are identified as complement receptors, reinforcing the involvement of complement signaling in this network. (Figure 6C,D). These complement and integrin dysregulations mirror immune suppression patterns in smoking, with shared epigenetic modifications affecting cancer-related genes like *HIC1*, though vaping avoids certain methylation changes (e.g., cg05575921) seen in smoking [43,44].

### 2.5. Immune Infiltration Analysis

**Figure 7:**
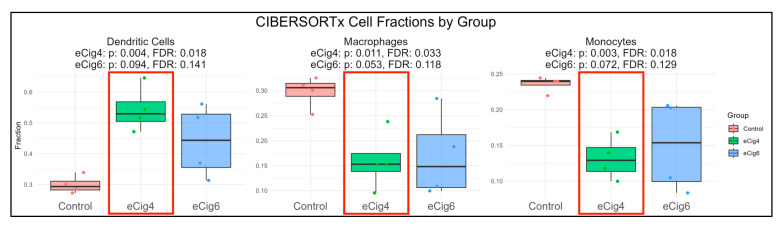
Immune infiltration differences among eCig4-induced IUGR (green), eCig6-induced PE (blue), and healthy control pregnancies (red). Cell types with statistically significant differences (FDR < 0.05) are outlined in red. Dendritic cells show significant changes in the eCig4 group (*p* = 0.040, FDR = 0.018) but not in eCig6 (*p* = 0.094, FDR = 0.141). Macrophage infiltration is significantly altered in eCig4 (*p* = 0.011, FDR = 0.033) and marginal in eCig6 (*p* = 0.053, FDR = 0.118). Monocytes are significantly increased in eCig4 (*p* = 0.003, FDR = 0.018), but not in eCig6 (*p* = 0.072, FDR = 0.129).

Our transcriptomic overlap analysis (Figure 4A) identified numerous immune-related DEGs shared between the eCig4 vs. Control and eCig6 vs. Control groups, and pathway enrichment results further emphasized immune-associated signaling (Figure 6A). These findings suggested that immune dysregulation might represent a key downstream consequence of eCig exposure. To determine whether these transcriptomic signatures corresponded to changes in immune cell composition, we applied CIBERSORTx to deconvolute immune cell signals in placental samples. This analysis revealed increased signaling of dendritic cells (FDR = 0.018) and decreased macrophages (FDR = 0.033) and monocytes (FDR = 0.018) in the eCig4-induced IUGR model, whereas no statistically significant differences were observed in the eCig6-induced IUGR + PE model (Figure 7). These changes in dendritic cells, macrophages, and monocytes reflect immune suppression patterns similar to those in smoking-exposed placentas, though vaping may induce unique epigenetic modifications and less severe DNA damage [43,44,46,47].

## 3. Discussion

We demonstrated that eCig exposure, whether before or after SA invasion in a C57BL/6 mouse model, induces similar gestational symptoms but produces distinct gene regulatory profiles. Exposure from E12.5 to E18.5 (eCig-6d), prior to SA invasion, induced both IUGR and PE-like symptoms. In contrast, exposure from E14.5 to E18.5 (eCig-4d), following SA invasion, resulted only in IUGR-like features without PE symptoms. In the eCig-4d vs. Healthy comparison, we observed downregulation of collagen-associated genes (*COL1A1*, *COL1A2*, *COL3A1*, *COL4A5*, *COL4A6*, *COL6A1*, *COL6A3*, *COL15A1*) and vasoactive genes (*NOS1*, *NOS2*, *NOS1AP*, *ARG1*), along with upregulation of inflammatory genes (*NR4A1*, *NR4A2*, *FOSB*). Conversely, eCig-6d vs. Healthy comparison shows downregulation of the complement system–associated genes (*C1qA–C*, *ITGAM*, *ITGB2*, *ITGAX*, *C5AR1*, *C3AR1*) and genes involved in granule structure formation.

### 3.1. DEG Analysis

Longer exposure time (eCig6 day) resulted in 64 significant DEGs compared to healthy control, while the shorter exposure time (eCig4 day) showed 429 significant DEGs. Although these findings may seem non-intuitive, one possible explanation is that when exposure begins before SA invasion (eCig6 day), the cellular environment may have time to adapt to the harmful effects of vaping eCigs. In contrast, initiating exposure after SA invasion (eCig4 day) occurs in a more established vascular environment, amplifying the detrimental impact. However, this increase in DEGs did not correspond to worsened gestational symptoms. We therefore speculate that a subset of these affected gene products may contribute to compensatory responses that could partially mitigate the effects of eCig exposure.

Our observation of fewer DEGs in the longer eCig-6d exposure, potentially indicating cellular adaptation, aligns with studies on placental resilience to environmental stressors. For instance, in models of chronic hypoxia or maternal smoking, prolonged exposure can trigger compensatory transcriptomic shifts that mitigate initial damage, though at the cost of sustained inflammation or fibrosis [48]. Similarly, temporal RNA-seq analyses in human PE placentas have shown that early gestational insults lead to broader DEG profiles compared to late-onset ones, supporting our hypothesis that eCig-4d exposure in a more established vascular environment amplifies acute responses [49]. However, discrepancies exist; some reports indicate diverse DEG profiles within PE patients, highlighting the need for time-course studies to clarify these dynamics [50]. 

Notably, since temporal progression data are lacking in these experiments, it is possible that the number of DEGs in eCig6 may have exceeded those in eCig4 at an earlier stage but were subsequently ameliorated by E18.

### 3.2. eCig4-Induced IUGR Model

Comparing eCig-4d vs. healthy controls induced upregulation of genes involved in NF-κB signaling, alongside downregulation of genes associated with endothelial vasoconstrictive function, weakened complement system initiation, and collagen formation pathways.

Specifically, we observed elevated *NR4A1*, *NR4A2*, and *FOSB* gene expression as key transcriptional regulators within the NF-κB and AP-1 pathways. Among them, NR4A1 and NR4A2 are nuclear receptors expressed in syncytiotrophoblasts, and are known to induce apoptosis, facilitate embryo adhesion, and regulate the timing of spontaneous term delivery [51]. Their upregulation in our study is consistent with findings in gestational diabetes and our earlier conclusions demonstrating eCig exposure induced apoptotic effects [52]. In line with this, *NR4A1* has been identified as a primary inflammatory modulator in macrophages via NF-κB signaling [52]. *FOSB*, a component of the AP-1 complex, was also upregulated and has previously been reported in PE, potentially through activation of the JNK/AP-1 pathway [53,54]. Heightened expression of these genes provides at least some support for the occurrence of immune tolerance dysfunction.

This NF-κB activation mirrors findings in human PE cohorts, where aberrant NF-κB signaling in trophoblasts promotes pro-inflammatory cytokine release and apoptosis, exacerbating placental dysfunction [55]. For example, USP17-mediated deubiquitinating of HDAC2 has been shown to modulate NF-κB in PE, leading to similar inflammatory profiles as observed here [56]. Furthermore, our downregulation of immune markers like ITGB2 and ITGAM, indicative of impaired M2 macrophage polarization, resonates with studies linking reduced anti-inflammatory phenotypes to IUGR, where monocyte/macrophage imbalances contribute to vascular remodeling failures [57]. These parallels suggest that eCig-4d exposure may recapitulate human PE immune dysregulation, though our model shows collagen downregulation rather than the fibrosis often reported in chronic PE [22,58].

Concomitantly, we observed downregulation of *NOS1*, *NOS2*, *NOS1AP*, and *ARG1*, which are critical to nitric oxide (NO) synthesis. Reduced expression of these genes leads to impaired NO production, as previously documented in IUGR cases where endothelial NO synthase activity and L-arginine transport are diminished [22]. This impairment may contribute to increased vasoconstriction and reduced placental perfusion. Our findings are aligned with previous proteomic studies examining placental development from E5 to E20, reinforcing the role of disrupted NO signaling in eCig-induced vascular dysfunction [59]. Impaired NO signaling aligns with mitochondrial dysfunction from oxidative stress, a shared effect of smoking and vaping, though vaping induces less DNA damage and unique methylation patterns distinguishing it from smoking [25,43,44]. Vaping’s nicotine content may also increase thrombus formation, potentially worsening placental vascular outcomes [45]. Impaired NO via NOS1/2 and ARG1 downregulation is corroborated by recent reviews linking diminished NO synthase to hypoperfusion and IUGR [60]. Recent ovine and murine studies emphasize time-sensitive NO protection in late gestation, supporting our post-SA invasion phenotype [61].

In addition to endothelial disruption, eCig-4d exposure altered immune-related gene expression. ITGB2 (CD18), a beta-2 integrin and subunit of complement receptor 4, was significantly downregulated. CD18 deficiency has been shown to impair monocyte proliferation during infection, and its reduced expression here may reflect compromised immune cell responsiveness [62]. Similarly, ITGAM (CD11b), a surface marker associated with monocyte/macrophage M2 polarization, was downregulated, suggesting a shift away from anti-inflammatory M2 phenotypes and potentially contributing to immune intolerance [62].

### 3.3. eCig6-Induced IUGR + PE

Comparing eCig-6d vs. healthy control samples resulted in the downregulation of several genes associated with the complement system, including *C1qA–C*, *ITGAM*, *ITGB2*, *ITGAX*, *C5AR1*, and *C3AR1*, as well as genes involved in granule structure formation.

Besides serving as an initiator of the classical complement cascade, C1q is also expressed by extravillous trophoblasts and decidual endothelial cells. It acts as a recognition molecule during SA invasion. C1q deficiency has been linked to shallow trophoblast invasion and impaired SA remodeling, which aligns with our findings [29].

The downregulation of *ITGAM* and *ITGAX* genes. ITGAX heterodimerize with ITGB2 to form complement receptors CR3 (CD11b/CD18) and CR4 (CD11c/CD18), suggests impaired leukocyte adhesion and migration [63]. This is concerning, as these integrins are critical for neutrophil trafficking and immune surveillance in the maternal–fetal interface. Moreover, dysfunction of CR3 and CR4 compromises the clearance of placental microparticles, apoptotic cells, and inflammatory debris, potentially contributing to an inflammatory placental environment [63,64].

Notably, ITGAM (CD11b) and ITGB2 (CD18) are stored in neutrophil secondary granules and translocated to the cell surface upon activation, where they facilitate adhesion and phagocytosis via CR3 [64,65,66,67]. Though our immune filtration analysis did not show the weakening signals of neutrophils, their reduced expression suggests diminished neutrophil responsiveness and may impair the resolution of inflammation during late gestation. Complement and integrin downregulation suggest immune suppression similar to smoking, with overlaps in epigenetic alterations (e.g., cg05575921 demethylation specific to smoke but not vaping) [43,44]. These effects may impair placental immune surveillance, consistent with smoking-related placental dysfunction [46].

Complement dysregulation here mirrors PE/IUGR pathways, where C1q/C3 imbalances impair trophoblast invasion and debris clearance [29,68]. eCig use is linked to adverse outcomes like low birth weight, potentially via similar immune disruptions [69]. Downregulated integrins (*ITGAM*, *ITGB2*) suggest leukocyte migration defects, consistent with recent findings on eCig-induced inflammation impairing fetal lung immunity [70]. Weakened neutrophil responsiveness may hinder inflammation resolution, paralleling IUGR neutrophil trafficking issues, though our bulk RNA-seq limits cell-specific insights [65].

### 3.4. Limitations

Bulk RNA-seq data should not be used to conclude the temporal progression of biological processes. Regulation of the transcripts usually lags behind the synthesis of proteins, sometimes even displays inverse relationships in quantity. On the other hand, in this study, we applied a weight filter of 0.9 to the KBoost results, which filters out more than 99% of the regulation pairs. Biologically significant information could have been discarded in the process. Moreover, single-cell RNA-seq or spatial transcriptomics could provide finer resolution in future studies. Another limitation in our study is that the nose-only eCig exposure protocol may not fully replicate human vaping patterns, which vary in frequency, nicotine concentration, and flavor additives. Finally, while the C57BL/6 mouse model recapitulates aspects of PE and IUGR, differences in placental structure and physiology between mice and humans (e.g., shallower trophoblast invasion in humans) may limit direct translatability to clinical settings. Our nose-only eCig model aligns with conditions for valid animal translation to human vaping, avoiding overheating [71]. However, bulk RNA-seq limits cell-specific insights into epigenetic changes differentiating smoking (greater harm) from vaping, particularly in placental contexts.

Overall, our molecular profiles reinforce eCig risks in pregnancy, aligning with recent population studies showing associations with preterm birth and small for gestational age [13]. Targeting NF-κB or complement (e.g., via inhibitors) holds promise, as demonstrated in PE models [72]. Future human cohort studies should validate these, addressing gaps in flavor-specific effects like menthol, which exacerbate embryonic stress [73,74].

## 4. Materials and Methods

### 4.1. Animal Husbandry and eCig Exposure Protocol

C57BL/6 mice were sourced from Jackson Laboratory (Bar Harbor, ME, USA). To control potential environmental influences on study outcomes, all mice were housed in the same room with a 12 h light/dark cycle, consistent temperature, humidity, and noise levels. Handling was minimized and performed by the same personnel at consistent times to reduce stress-induced variability in blood pressure or fetal growth. Diet (standard rodent chow) and water were provided ad libitum. Following confirmation of pregnancy, mice were subjected to eCig using a nose-only inExpose system (Scireq, Montreal, Canada). Exposures commenced at embryonic day 12.5 (E12.5, before spiral artery invasion) or E14.5 (after spiral artery invasion had started) and continued until E18.5. For eCig, the system delivered a single puff per minute over a daily 30 min session, buffering the vapor so mice received an 80mL continuous flow. The eCig liquids were cinnamon-flavored with a nicotine concentration of 6 mg/mL (propylene glycol/vegetable glycerin base, 50:50 ratio). Each 30 min session delivered approximately 0.48 mg nicotine per mouse, based on puff volume (80 mL/puff) and aerosol efficiency (~20% deposition rate in nose-only systems) [18]. Mice were divided into four groups (*n* = 6 per group): control (room air, control), eCig exposure for six days (eCig-6d), and eCig exposure for four days (eCig-4d). At E18.5, necropsies were performed, and fetal and placental weights were recorded. Urine samples were collected to assess proteinuria, and placental tissues were immediately frozen in liquid nitrogen for subsequent protein analysis.

### 4.2. Blood Pressure Assessment

Systolic and diastolic blood pressure were measured non-invasively using the CODA tail-cuff system (Kent Scientific, Torrington, CT, USA), which is equipped with an automated occlusion cuff and a warming platform. Mice were gently restrained in a clear, medium-sized cylindrical holder with an adjustable headpiece (Kent Scientific) for approximately five minutes during measurements. Blood pressure was recorded every other day on a heated pad to maintain consistent body temperature and assess potential hypertensive effects in exposed and control groups.

### 4.3. Proteinuria Analysis

To evaluate proteinuria as an indicator of preeclampsia, urine was collected at necropsy and analyzed using a colorimetric dipstick method. The dipstick results were categorized as negative, trace, +1 (30 mg/dL), +2 (100 mg/dL), +3 (300 mg/dL), or +4 (≥2000 mg/dL), with +3 and +4 indicating significant proteinuria consistent with preeclampsia. An Albuminuria Fluorometric Assay Kit (MyBioSource, San Diego, CA, USA) was used for quantitative validation to confirm dipstick findings.

### 4.4. RNA Extraction, Library Preparation, and Sequencing

Total RNA was extracted from the placental tissue of each mouse (*n* = 6) using the Direct-zol RNA MiniPrepPlus Kit with TRI Reagent (Zymo Research, Irvine, CA, USA, Cat No. R2052). Placental samples were homogenized in TRI Reagent at maximum speed, followed by centrifugation to collect the supernatant. The supernatant was mixed with an equal volume of 100% ethanol and loaded onto a Zymo-Spin IICR column. DNase I treatment was applied per the manufacturer’s guidelines, and subsequent column washes were performed as instructed. RNA was eluted in 30 μL of DNase/RNase-free water and stored at −80 °C until further processing. For library preparation, mRNA was isolated using poly-dT oligo-conjugated magnetic beads to facilitate cDNA synthesis. First-strand cDNA was generated with random hexamer primers, followed by second-strand synthesis, end polishing, A-tailing, and ligation of Illumina adapters. Libraries underwent size selection, amplification, and purification steps. Library quantification was performed using a Qubit fluorometer (Thermo Fisher Scientific, Waltham, MA, USA) and qPCR, with size distribution assessed via a Bioanalyzer (Agilent Technologies, Santa Clara, CA, USA). Paired-end sequencing of the normalized, pooled, and barcoded libraries was conducted on an Illumina NovaSeq instrument (Novogene, Sacramento, CA, USA). Batch effects were minimized by performing RNA extraction, cDNA synthesis, library preparation, and sequencing from all samples simultaneously at each step.

### 4.5. Statistical Evaluation

Differences in fetal weight, placental weight, proteinuria, and blood pressure were assessed for statistical significance using an unpaired Student’s *t*-test, with a *p*-value threshold of <0.05 indicating significance. Sample size (*n* = 6 per group) was determined based on prior mouse models of PE and IUGR, where similar group sizes have detected significant physiological and transcriptomic differences [75]. For the RNA-sequencing data, differential gene expression was evaluated using the edgeR package, which applies a negative binomial distribution model. Multiple comparison adjustments were made using the false discovery rate (FDR) correction, with significance defined as FDR < 0.05.

### 4.6. RNA-Seq Analysis

Bulk RNA-seq data were processed using the RASflow pipeline (publication version: 20 April 2020), which automates key steps of the workflow. Briefly, raw reads were trimmed using Trim Galore (https://www.bioinformatics.babraham.ac.uk/projects/trim_galore/ (accessed on 11 November 2019) (Version 0.6.5), and quantified at the transcript level with Salmon (Release: v1.10.1) [76,77]. Gene-level counts were aggregated using tximport, and differential expression analysis was performed with edgeR (Version 4.6.3) for three comparisons: (1) eCig4 vs. control, (2) eCig6 vs. control, and (3) eCig6 vs. eCig4. DEGs were identified using an FDR-adjusted *p*-value < 0.05 [78]. Pathway enrichment was conducted using signaling pathway impact analysis (SPIA) via the R EnrichmentBrowser package (Version 2.38.1), with pathway annotations from KEGG and Reactome (Release 2022) using human ortholog genes (Release version Gene 115) [37,38]. The raw gene count matrix was used with the KBoost algorithm (Version 1.16.0) to identify the most influential upstream regulators [34]. Pathway analysis heatmaps were generated based on the significantly enriched pathways for each condition (weight > 0.9; eCig6, weight > 0.5 due to the small number of DEGs, FDR *p*-value < 0.05). Although pathways contain many genes, only genes that were significantly differentially expressed within the significant pathways were visualized to improve clarity. The GRN was then constructed with protein–protein interaction data from the STRING database (Version 12) [79]. Immune cell deconvolution was performed using CIBERSORTx (year 2025) to estimate each sample’s abundance of immune populations [38]. A mouse-specific signature matrix was used as the reference, and the raw count matrix was uploaded through the CIBERSORTx web interface [72]. Batch correction was enabled using B-mode, and the number of permutations was set to 500. FDR-adjusted *p*-values (Benjamini–Hochberg) shown in boxplots were corrected based on the number of immune cell types displayed.

## 5. Conclusions

eCig exposure induces immune suppression and mitochondrial dysfunction via dysregulated complement, inflammatory, and NOS pathways, with similarities to traditional smoking in gene expression and epigenetic changes linked to cancer/immune risks, though vaping poses lower overall harm [43,44,45]. In contrast, eCig-6d exposure leads to downregulation of complement-related genes and granule-associated components, suggesting weakened neutrophil responsiveness and compromised inflammatory clearance at the maternal–fetal interface.

## Figures and Tables

**Table 1:** Statistics of different genes from Section 2.3 in the eCig4-Induced Model. The table shows the gene symbol, gene description, their respective log_2_ fold change (log_2_FC), and their false discovery rate (FDR).

Gene	Description	log_2_FC	FDR
ARG1	Arginase 1	−2.45	1.39 × 10^−6^
C1QA	Complement c1q a chain	−1.5	4.10 × 10^−13^
C1QC	Complement c1q c chain	−1.19	4.77 × 10^−8^
C5AR1	Complement c5a receptor 1	−1.1	1.47 × 10^−3^
COL15A1	Collagen type xv alpha 1 chain	−1.4	4.24 × 10^−7^
COL1A1	Collagen type i alpha 1 chain	−1.75	4.12 × 10^−4^
COL1A2	Collagen type i alpha 2 chain	−1.33	3.46 × 10^−3^
COL3A1	Collagen type iii alpha 1 chain	−1.25	1.13 × 10^−5^
COL4A5	Collagen type iv alpha 5 chain	−1.55	8.32 × 10^−4^
COL4A6	Collagen type iv alpha 6 chain	−2.67	1.51 × 10^−4^
COL6A1	Collagen type vi alpha 1 chain	−1.29	3.25 × 10^−3^
COL6A3	Collagen type vi alpha 3 chain	−1.12	0.014
FOSB	Fosb proto-oncogene, ap-1 transcription factor	0.94	3.25 × 10^−3^
HCK	Hck proto-oncogene, src family tyrosine kinase	−1.14	1.71 × 10^−3^
ITGAM	Integrin subunit alpha m	−1.49	1.80 × 10^−7^
ITGB2	Integrin subunit beta 2	−1.53	1.75 × 10^−4^
NOS1	Nitric oxide synthase 1	−0.74	0.011
NOS1AP	Nitric oxide synthase 1 adaptor protein	1.12	0.021
NOS2	Nitric oxide synthase 2	0.63	0.042
NR4A1	Nuclear receptor subfamily 4 group a member 1	1.36	0.037
NR4A2	Nuclear receptor subfamily 4 group a member 2	1.49	1.71 × 10^−3^
TYROBP	Transmembrane immune signaling adaptor	−0.98	8.49 × 10^−5^

**Table 2:** Statistics of different genes from Section 2.4 in the eCig6-Induced Model. The table shows the gene symbol, gene description, their respective log_2_ fold change (log_2_FC), and their false discovery rate (FDR).

Gene	Description	log_2_FC	FDR
C1QA	Complement c1q a chain	−1.12	9.75 × 10^−6^
C1QB	Complement c1q b chain	−1.15	3.94 × 10^−7^
C1QC	Complement c1q c chain	−1.15	2.26 × 10^−7^
C3AR1	Complement c3a receptor 1	−0.81	0.029
C5AR1	Complement c5a receptor 1	−1.56	1.12 × 10^−6^
ITGAM	Integrin subunit alpha m	−1.35	3.94 × 10^−6^
ITGAX	Integrin subunit alpha x	−1.36	0.010
ITGB2	Integrin subunit beta 2	−1.47	1.92 × 10^−9^

## Data Availability

The original contributions presented in the study are included in the article. Processed reads (.sf) are available at https://zenodo.org/records/16914711 (accessed on 8 October 2025). https://doi.org/10.5281/zenodo.16914711 Count matrix included. Preprocessed raw reads can be found at https://zenodo.org/records/16914793 (accessed on 8 October 2025) and https://zenodo.org/records/16915391 (accessed on 8 October 2025).
